# Multicenter survey on implantable collamer lens dislocation

**DOI:** 10.1371/journal.pone.0264015

**Published:** 2022-02-14

**Authors:** Takashi Kojima, Yoshihiro Kitazawa, Tomoaki Nakamura, Kazutaka Kamiya, Kazuo Ichikawa, Akihito Igarashi, Kimiya Shimizu

**Affiliations:** 1 Nagoya Eye Clinic, Nagoya, Japan; 2 Sapia Tower Eye Clinic, Tokyo, Japan; 3 Department of Ophthalmology, University of Kitasato School of Medicine, Kanagawa, Japan; 4 Chukyo Eye Clinic, Nagoya, Japan; 5 Department of Ophthalmology, Sanno Hospital, Tokyo, Japan; Saarland University, GERMANY

## Abstract

This study aimed to investigate the incidence, patient background, and postoperative prognosis of implantable collamer lens (ICL) dislocation. We retrospectively reviewed all cases of ICL dislocation at four major refractive surgery centers in Japan until December 2019. The incidence, patient background, cause of dislocation, complications of repositioning surgery, and postoperative visual function were investigated. Seven ICL dislocations [0.072% of total ICL-implanted eyes (9775 eyes)] occurred at an average of 28.6 months (11–82 months) postoperatively. All patients were male. Five eyes were injured during sports activities, one due to a fall from a bicycle, and another due to ocular blunt trauma caused by a mortuary tablet. Two patients had re-dislocation in the same eye. Retinal detachment occurred after repositioning surgery in one patient, and scleral buckling surgery was performed without ICL removal. ICL dislocation is a rare complication of ICL surgery; repositioning surgery is effective, but retinal complications may occur.

## Introduction

Implantable collamer lenses (ICL) have been found to be effective and safe for the correction of mild to moderate and high myopia [1–4]. Toric ICL implantation is more effective than laser in situ keratomileusis (LASIK) for correcting high astigmatism [5]. Laser corneal refractive surgery is contraindicated in eyes with thin or abnormal corneas, such as those with keratoconus, and ICL implantation has been confirmed to be highly effective and safe in patients with mild keratoconus or borderline pachymetry due to its minimal impact on the cornea [6–8].

Postoperative complications rarely occur after ICL implantation, although cataract formation, pigment dispersion, intraocular inflammation, toxic anterior segment syndrome, and toric ICL rotation have been reported [9–12]. Laser corneal refractive surgery, such as LASIK, may lead to ocular trauma, resulting in flap displacement [13, 14]. Therefore, surface ablation surgeries, such as photorefractive keratectomy, laser-assisted subepithelial keratectomy, and epi-LASIK, are recommended for patients with hobbies or occupations that are associated with a risk of direct impact to the eye. In general, ICL implantation is performed through a small incision, and since there is no flap formation in the cornea as in LASIK, it seems to be more resistant to trauma.

Several studies have reported on ICL dislocation due to blunt ocular trauma after ICL surgery [15–20]. Most of these reports were single-center case reports. This study aimed to retrospectively review cases of ICL dislocation that occurred at four major refractive surgery centers in Japan and determine the incidence of ICL dislocation, patient background, and prognosis after ICL repositioning surgery.

## Materials and methods

The multicenter study was conducted at Kitasato University, Nagoya Eye Clinic, Kobe Kanagawa Eye Clinic, and Sanno Hospital in Japan. We retrospectively compiled and reviewed all cases of ICL dislocation encountered at these institutions until December 2019.

The incidence of ICL dislocation, preoperative patient background, cause of ICL dislocation, timing of ICL repositioning surgery, and postoperative course were investigated. Additionally, we evaluated the safety and efficacy of ICL repositioning surgery by comparing the uncorrected and corrected distance visual acuity at the last follow-up before ICL dislocation with those after ICL repositioning surgery.

Vault height, distance between crystalline lens and ICL, were also recorded from the patients’ chart. A vault of 1/2 to 3/2 (250–750 μm) of the corneal thickness (CT) was considered a moderate vault.

Intraoperative and postoperative complications were also evaluated. This study was approved by the Institutional Review Board of the Nagoya Eye Clinic (#2020–44). The requirement for informed consent was waived by the Institutional Review Board.

## Results

### Incidence

**Fig 1:**
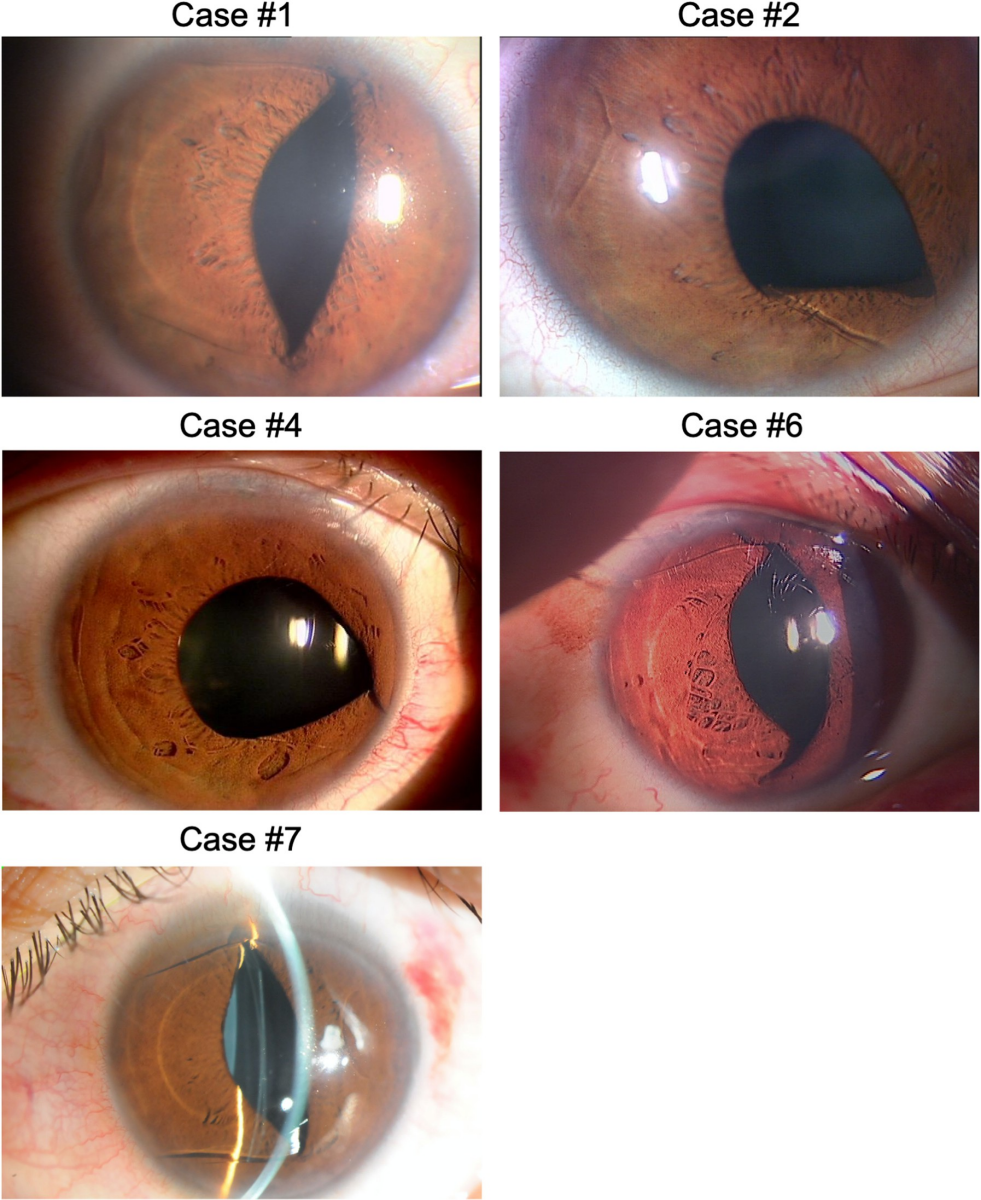
Representative slit-lamp findings of five patients with implantable collamer lens (ICL) dislocation. Case #1, ICL dislocation caused by another player’s hand hitting the right eye during futsal match. The superotemporal and inferotemporal haptics have prolapsed into the anterior chamber. Case #2, ICL dislocation caused by a futsal ball hitting the right eye. This case is a recurrent ICL dislocation of case #1. Case #4, ICL dislocation was caused by a futsal ball hitting the right eye. The inferonasal haptic has prolapsed into the anterior chamber. Case #6, ICL dislocation caused by a mortuary tablet hitting the right eye while working at a temple. The inferotemporal and superotemporal haptics have prolapsed into the anterior chamber. Mild anterior chamber inflammation was noted at the first visit after ICL dislocation, and retinal detachment was found at 8^th^ day of ocular injury. Case #7, ICL dislocation caused by another player’s elbow hitting the right eye during basketball match. The inferotemporal and superotemporal haptics have prolapsed into the anterior chamber.

The incidence of ICL dislocation was 0.072%; ICL dislocation occurred in seven of the 9775 eyes that underwent ICL surgery at the four centers. Fig 1 shows representative slit-lamp photographs of the patients at the first visit after ICL dislocation.

### Patient background

The background of the patients and the causes of ICL dislocation are summarized in Table 1. The mean age at the time of dislocation was 29.2±6.0 years, and all patients were men. Two patients experienced ICL dislocation twice in the same eye, with the second dislocation occurring 11 and 15 months after the first dislocation. The mean preoperative equivalent spherical power of the eyes with ICL dislocation was -9.0 ± 4.2 D. Six of the seven eyes had high myopia (>6 D).

**Table 1:** Patient demographic information and cause of ocular trauma.

Case	Age (years)/Sex/Eye	ICL power (Sphere, Cylinder)	ICL size (mm)	IOP (mmHg)	Cause of trauma (time after ICL implantation)	Post-trauma slit lamp examination findings	Time of repositioning surgery (days after eye injury)
#1	39/M/OD	-13.5, 3.5	12.6	18	Blunt ocular trauma from another player’s hand during a futsal match (11 months)	Prolapse of the superotemporal and inferotemporal haptics into the AC	same day
						Mild AC inflammation	
#2	39/M/OD	-13.5, 3.5	12.6	19	Blunt ocular trauma from another player’s foot during a futsal match (2 years and 2 months)	Prolapse of the inferonasal haptic in the AC	1 day
						Mild AC inflammation	
#3	25/M/OD	-16.5, 1.5	13.2	24	Direct hit from a futsal ball (10 months)	Prolapse of the inferonasal haptic into the AC	3 days
#4	26/M/OD	-16.5, 1.5	13.2	18	Direct hit from a futsal ball (21 months)	Prolapse of the inferonasal haptic into the AC	5 days
#5	28/M/OS	-17.5, 2	12.6	12	Blunt ocular trauma from a pole on the road while riding a bicycle (1 year and 2 months)	Prolapse of the inferonasal haptic into the AC	2 days
#6	24/M/OD	-16	12.6	15	Blunt ocular trauma from a Mortuary tablet while working at a temple (7 years and 10 months)	Prolapse of the inferotemporal and superotemporal haptics into the AC	3 days
						Mild AC inflammation	
#7	30/M/OD	-14.5, 1.5	13.2	13	Blunt ocular trauma from another player’s elbow while playing basketball (3 years)	Prolapse of the superotemporal and inferonasal haptic prolapse into the AC	same day

M: male, OD: oculus dextrus, OS: oculus sinister, ICL = implantable collamer lens, IOP = Intraocular pressure, AC = anterior chamber.

### Causes of ICL dislocation

All patients experienced ICL dislocation due to blunt ocular trauma. Four of the eyes were injured during a futsal game. Among them, two were injured by a direct hit from a ball, one was injured by another player’s hand, and one was injured by another player’s foot. Of the remaining three eyes, one was injured by another player’s elbow during a basketball game, one was injured by a roadside pole during a fall from a bicycle, and one was injured when the patient, a priest, was hit in the eye by a mortuary tablet.

### Timing of ICL repositioning surgery

On average, ICL repositioning surgery was performed 2.14±1.68 days (0–5 days) after injury.

### Surgical technique

Five and two patients underwent surgery under topical anesthesia alone and topical anesthesia with intracameral anesthesia, respectively. A 1-mm incision was made, viscoelastic material was injected into the anterior chamber through this incision, and the dislocated haptics were repaired using an ICL manipulator. Finally, irrigation and aspiration were performed to remove the viscoelastic material from the anterior chamber, and the surgery was completed. Antibiotics and steroid eye drops were administered for 2 weeks to 1 month postoperatively.

### Postoperative changes in the vault

In 5 of the 7 eyes, the vault was measured by slit lamp microscopy; the two remaining eyes were assessed by anterior segment optical coherence tomography. Preoperatively, three eyes had a high vault and four had a moderate vault. The changes in vault from before trauma to after ICL repositioning surgery were as follows: case #1, 0.9 mm to 0.82 mm; case #2, from 0.82 mm to 0.79 mm; case #3, from 1.5 CT to 1.5CT; case #4, from 1.5 CT to 1.5 CT; case #5, from 1.25 CT to 1 CT; case #6, from 1 CT to 1 CT; and case #7, from 2 CT to 1.5 CT.

### Postoperative course

The results of ICL repositioning surgery are summarized in Table 2. There was no significant difference in the mean uncorrected distance visual acuity (logMAR) between the last follow-up before ICL dislocation (-0.04±0.17) and 3 months after ICL repositioning surgery (-0.11±0.16, p = 0.81). Similarly, there was no significant difference in the mean corrected distance visual acuity between the last follow-up before ICL dislocation (-0.19±0.05) and 3 months after ICL repositioning surgery (-0.18±0.09, p>0.999).

**Table 2:** Clinical outcomes after ICL repositioning surgery.

Case	Final visit before trauma					Three months after repositioning surgery					
	UDVA (logMAR)	CDVA (logMAR)	IOP (mmHg)	Vault	ECD (cells/mm^2^)	UDVA (logMAR)	CDVA (logMAR)	IOP (mmHg)	Vault	ECD (cells/mm^2^)	Complications
#1	-0.176	-0.301	15	0.9 mm	3125	-0.176	-0.176	13	0.82 mm	3001	None
#2	-0.176	-0.176	13	0.82 mm	3001	-0.301	-0.079	17	0.79 mm	3050	None
#3	-0.079	-0.176	22	1.5 CT	2976	-0.079	-0.176	21	1.5 CT	2985	None
#4	-0.079	-0.176	21	1.5 CT	2985	-0.079	-0.079	17	1.5 CT	2849	None
#5	-0.176	-0.176	15	1.25 CT	3003	-0.079	-0.176	17	1 CT	2958	None
#6	0.222	-0.176	15	1 CT	2808	0.097	-0.301	12	1 CT	2739	RD 8 days after injury
											Scleral buckling
											Pigment deposition on the ICL surface
#7	-0.176	-0.176	18	2 CT	2854	-0.301	-0.301	12	1.5 CT	-	Pigment deposition on the ICL surface

ICL = implantable collamer lens, UDVA = uncorrected distance visual acuity, CDVA = corrected distance visual acuity, IOP = Intraocular pressure, ECD = corneal endothelial cell density, CT = corneal thickness, RD = retinal detachment.

There was no significant difference between the mean spherical equivalent power at the last follow-up before ICL dislocation (-0.32 ± 0.51 D, +0.25 to -0.75 D) and at 3 months after ICL repositioning surgery (-0.25 ± 0.46, +0.375 to -0.75 D; p = 0.484). The mean absolute change in spherical equivalent power between these two time points was 0.29 ± 0.20.

There was no significant difference between the mean corneal endothelial cell density between the last follow-up before ICL dislocation (3011±107/mm^2^) and 3 months after ICL repositioning surgery (2955±149/mm^2^, p = 0.2969).

### Postoperative complications

All the patients had an intraocular pressure of < 21 mmHg on the first postoperative day. Mild anterior chamber inflammation was observed, but it disappeared within 1 week of surgery. At 3 months postoperatively, mild pigmentation was observed on the ICL surface in two patients. One patient showed mild commotio retinae after injury, which resolved within two days.

One patient, who was a priest and experienced injury when a mortuary tablet hit him in the eye, developed retinal detachment 8 days after injury and underwent scleral buckling surgery without ICL removal. Three months after scleral buckling surgery, his uncorrected distance visual acuity (logMAR) was 0.046 and his corrected distance visual acuity was 0.176 (sphere: -0.25, cylinder: -0.75, axis: 180).

## Discussion

Here, we investigated the incidence, patient background, and postoperative prognosis of ICL dislocation at four major refractive surgery centers in Japan. Seven ICL dislocations occurred during the study period. The incidence of ICL dislocation was 0.072%, suggesting that it is very rare. Studies have reported incidence rates of 0.085% and 0.012% for flap displacement due to trauma, a late postoperative complication of LASIK [21, 22]; these rates are similar those of ICL dislocation.

Most of the patients in this study were young (mean age: 29.2 years), and all of them were men, which may be related to the high proportion of injuries occurring during sports such as futsal and basketball.

Two patients experienced ICL dislocation twice in the same eye. A previous study reported on patients with recurrent ICL dislocation [18], one of whom was also included in this study. In this study, both cases of dislocation were caused by blunt trauma during a futsal game; in one case, both dislocations occurred due to the ball hitting the face at a close range, and in the other case, they occurred due to impact with another player’s hand or foot. Futsal is popular in Japan because it does not require a large stadium and can be played in a small space. Here, all futsal-related eye injuries occurred at night. Most futsal stadiums have lighting, but they are dimly lit at night. Additionally, the sympathetic dominant state of the player might have resulted in pupillary dilatation. Consequently, the patients might have had some degree of mydriasis at the time of trauma, and the eyes were injured in a situation where the ICL could easily be dislocated.

Five out of the seven cases of ICL dislocation in this study were caused by blunt ocular trauma during sports: four during futsal games and one during a basketball game. Considering that more than one million ICLs have been implanted worldwide, it is important to educate patients to use protective eyewear during ball games with a risk of blunt ocular trauma.

The vault is the distance between the anterior surface of the crystalline lens and the posterior surface of the ICL. In general, an ICL shows a high vault when its length is greater than the ciliary sulcus diameter. A high vault has been suggested to be a risk factor for pigment dispersion and angle-closure glaucoma [9, 10, 23, 24]. In this study, the vault was considered high when it was greater than 1.5 times the corneal thickness, and three patients (42.9%) showed a high vault at the last follow-up before ICL dislocation. In one eye, the vault was twice the corneal thickness. To date, it is unclear whether a high vault predisposes the ICL to trauma-induced dislocation. In patients with a high vault, the ICL footplate may be subjected to a strong compressive force, which may lead to trauma-induced ICL dislocation. Due to the small number of cases, we were not able to determine the relationship between a high vault and ICL dislocation, but this issue should be investigated in future studies with larger samples. In addition, in four of the seven eyes, the vault after ICL repositioning surgery was slightly lower than that before trauma. The ICL is implanted in the ciliary sulcus, and sulcus to sulcus diameter reportedly differs in the horizontal and vertical directions [25]. It is possible that the ICL repositioning surgery may have slightly altered the position of the ICL, or that the trauma caused changes in the ciliary sulcus diameter. Further studies are needed to verify whether these changes are clinically significant.

In this study, there was no significant difference in corneal endothelial cell density between the last follow-up before ICL dislocation and 3 months after ICL repositioning surgery, and no patient had major corneal endothelial cell damage. However, a case has been reported in which a dislocated ICL came into contact with the corneal endothelium, resulting in corneal endothelial cell damage that led to bullous keratopathy [17]; the patient underwent descemet stripping automated endothelial keratoplasty on the fifth day after injury. In our study, the ICL was repositioned an average of 2.1 days after injury. In the patient who underwent repositioning 5 days after injury, there was no contact between the ICL and corneal endothelium. If there is direct contact between corneal endothelial cells and the ICL, endothelial cell damage may worsen over time. Therefore, patients should be encouraged to seek medical attention as soon as possible after the injury, and repositioning surgery should be performed as early as possible.

One patient experienced retinal detachment on the eighth day after ICL repositioning surgery, and retinal surgery was required. The patient was a priest who had been injured when a wooden mortuary tablet hit his eyeball. He also had an eyelid laceration, leading us to believe that the external force of the eyeball strike was quite strong. Since he only had detachment of the peripheral retina, the ICL was not removed, and the retina was repaired with scleral buckling surgery alone. There was almost no decrease in corrected distance visual acuity or change in refraction postoperatively. In this case, a dilated fundus examination was not performed before ICL repositioning surgery. Another patient had mild commotio retinae after injury, which disappeared after 2 days. These cases suggest that retinal damage may occur in cases of blunt ocular trauma that is strong enough to cause ICL dislocation, and that it is important to monitor the retina carefully after injury.

In all seven patients, visual acuity and refraction recovered to the pre-trauma level. In the absence of other complications, ICL dislocation has a good prognosis, and visual function can be maintained with early repositioning surgery. Patient education is important to avoid repeated ICL dislocation.

## Supporting information

S1 Data (XLSX)
